# Selenium Attenuates HPV-18 Associated Apoptosis in Embryo-Derived Trophoblastic Cells but Not Inner Cell Mass In Vitro

**DOI:** 10.1155/2015/562567

**Published:** 2015-08-04

**Authors:** Jennifer A. Tolen, Penelope Duerksen-Hughes, Kathleen Lau, Philip J. Chan

**Affiliations:** ^1^Department of Gynecology and Obstetrics, 11370 Anderson Street, Suite 3950, Loma Linda University School of Medicine, Loma Linda, CA 92354, USA; ^2^Department of Basic Sciences, Center for Health Disparities and Molecular Medicine, 11021 Campus Street, Loma Linda University School of Medicine, Loma Linda, CA 92350, USA

## Abstract

*Objectives*. Human papillomaviruses (HPV) are associated with cell cycle arrest. This study focused on antioxidant selenomethionine (SeMet) inhibition of HPV-mediated necrosis. The objectives were to determine HPV-18 effects on embryonic cells and to evaluate SeMet in blocking HPV-18 effects. *Methods*. Fertilized mouse embryos were cultured for 5 days to implanted trophoblasts and exposed to either control medium (group 1), HPV-18 (group 2), combined HPV-18 and 0.5 *µ*M SeMet (group 3), or combined HPV-18 and 5.0 *µ*M SeMet (group 4). After 48 hrs, trophoblast integrity and, apoptosis/necrosis were assessed using morphometric and dual-stain fluorescence assays, respectively. *Results*. HPV-18 exposed trophoblasts nuclei (253.8 ± 28.5 sq·*µ*) were 29% smaller than controls (355.6 ± 35.9 sq·*µ*). Supplementation with 0.5 and 5.0 *µ*M SeMet prevented nuclear shrinkage after HPV-18 exposure. HPV-18 infected trophoblasts remained larger with SeMet supplementation. HPV-18 decreased cell viability by 44% but SeMet supplementation sustained cell viability. Apoptosis was lower when SeMet was present. HPV-18 decreased inner cell mass (ICM) viability by over 60%. *Conclusions*. HPV-18 decreased nuclear size and trophoblast viability but these effects were attenuated by the antioxidant SeMet. SeMet blocked HPV-18 associated apoptosis process in trophoblasts but not ICM cells suggesting involvement of different oxidative stress pathways.

## 1. Introduction

Human papillomaviruses (HPV) are classified group 1 human carcinogens (IARC, International Agency for Research on Cancer) extensively studied for their role in cervical cancer [1]. HPV are double-stranded DNA viruses with a well-defined affinity for epithelial cells. The high risk HPV serotypes 16 and 18 are responsible for 50–70% of all cases. HPV has been recovered more frequently in placentas obtained from patients with spontaneous preterm labor and spontaneous abortions than from term or elective abortions [2–6]. Gomez et al. [2] showed that HPV infection of trophoblasts induces cell death and reduces cell invasion, possibly placental invasion into the uterine wall. Preterm birth is the single largest direct cause of neonatal deaths, directly resulting in 35% of the world's 3.1 million deaths a year [7]. Recently, HPV has been shown to increase oxidative stress [8] that is associated with pregnancy complications, premature rupture of membrane, and preterm birth [9–11]. However, treatment modalities for HPV-mediated placental pathogenesis are lacking and studies are warranted.

The trace element selenium is a component of antioxidative selenoenzymes, Glutathione Peroxidase (GPx) and Thioredoxin Reductase (ThxRed), that decrease oxidative stress. Studies in BeWo and JEG-3 trophoblast cell lines provided evidence that inorganic sodium selenite and organic selenomethionine (SeMet) protected cells from oxidative stress [12, 13]. SeMet is a selenium-containing amino acid that upregulates the 2 selenoenzymes GPx and ThxRed responsible for depleting reactive oxygen species (ROS) and reactive nitrogen species (RNS) thereby reducing oxidative stress [13, 14]. The role of selenium compounds as anticancer agents has been reviewed [15, 16].

We hypothesized that treatment with antioxidant promoting SeMet would inhibit HPV-mediated embryonic cell necrosis. Recently, cultured trophoblast cells from implanted embryos were used as a model for HPV infectivity studies [17, 18]. In this study, the objectives were (1) to determine the effects of HPV-18 exposure on cultured cells from the trophoblast and inner cell mass (ICM) layers and (2) to evaluate the role of SeMet in preventing the damaging effects of HPV-18 on the ICM and trophoblast cells.

## 2. Materials and Methods

### 2.1. HeLa Cell Lysate Procedure

The preparation of HeLa cell lysates was as previously reported [17, 18]. HeLa cells (ATCC, American Type Culture Collection, Manassas, VA) derived from human cervical cell carcinoma contained multiple copies of HPV-18 integrated at chromosome 8, band q24 [19, 20]. The HeLa cells were cultured in Eagle Minimal Essential Medium (MEM, Invitrogen, Carlsbad, CA), supplemented with 10% fetal bovine serum (Invitrogen), 100 U/mL penicillin, 0.1 mg/mL streptomycin, and 0.25 *μ*g/mL amphotericin B (Sigma Aldrich Chemical Co., St. Louis, MO). The cells were passaged and processed after reaching 80% confluence [21]. The HeLa cells were centrifuge-washed at 300 ×g for 10 mins and the pellets of cells combined in a microfuge tube containing 0.4 mL of G-2 plus version 5 medium (G-2 v5, VitroLife, Englewood, CO). HPV-18 gene fragments were extracted from cells lysed using a sterile rod and pestle apparatus. The cell extract was stored frozen in cryovials at −23°C. The lysates were warmed to 37°C prior to their use in the mouse embryo experiments.

### 2.2. Culture of Pronuclear Mouse Embryo to the Implanted Stage

The procedure for culturing embryos to the implanted stage was as previously reported [22, 23]. Briefly, cryopreserved 1-cell mouse fertilized oocytes in cryostraws (Embryotech Laboratories Inc., Haverhill, MA) were thawed into a droplet of G-1 v5 medium (G-1 v5, VitroLife, Englewood, CO) in a Petri dish. The 1-cell fertilized embryos were washed through 2 additional droplets of G-1 v5 medium. The embryos were pooled at the center well of a double-well culture dish (Falcon 3037, Becton Dickinson, Franklin Lakes, NJ) containing 1 mL of G-1 v5 medium. Water was placed on the outer moat for humidity and each dish was placed in an incubator at 37°C, 5% CO_2_ in air mixture. After 3 days, the embryos were randomly divided into 4 groups and placed into 4 culture dishes containing G-2 v5 medium with more nutrients for blastocyst growth. Embryos that had not developed to the early blastocyst stage were not used. The embryos were further incubated for an additional 2 days until they reached the implanted stage.

A concentrated stock solution of the selenium-containing amino acid, selenomethionine (SeMet, Sigma Aldrich Chemical Co., St. Louis, MO), was dissolved in G-2 v5 medium and used in the preparation of the 0.5 and 5.0 *μ*M SeMet culture media. The 0.5 *μ*M concentration was based on a previous study in trophoblast cells [13]. Implanted embryos in each group were exposed to either control medium containing heat-inactivated HPV-18 HeLa lysate (10 *μ*L) (group 1), thawed HeLa cell lysate (10 *μ*L) containing active HPV-18 (group 2), combined HeLa lysate and 0.5 *μ*M SeMet medium (group 3), or combined HeLa lysate and 5.0 *μ*M SeMet medium (group 4). The cell cultures were further incubated for 48 hrs. Cell viability status, trophoblast nuclear size, and cell area were assessed as described below.

### 2.3. Dual Fluorescence Stain Analysis for Cell Status

The dual fluorescence stain method [18, 22] was used to distinguish viable, apoptotic, or necrotic trophoblast cells in each embryo. Briefly, a drop of bisbenzimide (5 *μ*L of 10 *μ*M, Hoechst 33342, Sigma Chemical Co., St. Louis, MO) was added to the implanted embryos and after 1 minute, 5 *μ*L of propidium iodide (32 *μ*M, Sigma Chemical Co., St. Louis, MO, dissolved in saline) was added to the embryos. After another minute, the culture medium with the stains was discarded and prewarmed culture medium (0.5 mL) was added to each dish. The culture dish was placed on the stage of an epifluorescence UV-microscope set at magnification of 500x (Nikon Optiphot, Nikon Instruments, Melville, NY) and images of the fluorescent colored cells in each embryo were captured on a digital camera. The percentages of viable, apoptotic, and necrotic cells of the ICM and trophoblast layers were determined for each embryo. In this study, a viable cell was defined as the capacity of the cell to exclude the fluorescent dyes and possessed a clear coloration. In contrast, an apoptotic (completely blue color) or necrotic (pink-red color) cell was identified by the cell capacity to absorb bisbenzimide or propidium iodide stain, respectively.

### 2.4. Trophoblast Morphology Stain Procedure

Trophoblast cells and inner cell mass nuclei and cytoplasm were stained using a sequential Fast Green FCF and Rose Bengal (Spermac Stain Enterprises Inc., Republic of South Africa) staining procedure [18, 23]. The procedure involved rinsing the implanted embryos with culture medium followed by fixation in 4% formalin (5 mins). The fixative was rinsed off with water and Spermac stain A (Rose Bengal mixture) was added (2 minutes). Stain A was rinsed off and stain B (Pyronin Y mixture) was added (1-minute staining) followed by rinsing and staining with stain C (Fast Green FCF-Janus Green mixture, 1 minute). The stained embryos were rinsed in water, air-dried, and stored in a dark drawer until the time of analyses.

### 2.5. Spectrophotodensitometry

Cell dimensions were measured using a spectrophotodensitometric method which facilitated the determination of embryo growth and migration [18]. The Spermac-stained inner cell mass and trophoblast cells were located on each slide using the Nikon Diaphot inverted microscope (400x magnification) and the images digitized and recorded. The preanalytical phase included using Adobe Photoshop software to standardize each image [24]. The image outline of each nucleus or the entire trophoblast cell was traced onscreen and measurements were determined using the Adobe Photoshop histogram function. The recorded data was entered into Microsoft Excel spreadsheets and analyzed.

### 2.6. Statistical Analysis

Data from the morphometric analyses of cell dimensions were presented as mean ± standard error of the mean (S.E.M.). For each treatment group, the mean dimension of the ICM or trophoblast nucleus and entire cell area were calculated and tested using ANOVA and significance of means was compared using the two-tailed Student's *t*-test. The numbers of viable, apoptotic, or necrotic trophoblast cells for each treatment group were tested using the two-tailed Mantel-Haenszel chi-square test statistics (http://OpenEpi.com/, Open Source Epidemiologic Statistics for Public Health, Emory University, Atlanta, GA). A value of *p* < 0.05 was considered significant.

## 3. Results

The nuclear size (Table 1) of HPV-18 exposed trophoblasts (253.8 ± 28.5 sq·*μ*) was 29% smaller (*p* < 0.03) than control trophoblasts (355.6 ± 35.9 sq·*μ*). The supplementation with 0.5 *μ*M SeMet significantly reversed (*p* < 0.005) the nuclear shrinkage in the HPV-18 exposed trophoblasts (401.9 ± 47.8 sq·*μ*). Interestingly, the addition of the higher concentration of SeMet (5.0 *μ*M) also prevented nuclear shrinkage in the HPV-18 infected trophoblasts but the effect was less pronounced (275.5 ± 23.1 sq·*μ*). A shrunken reduced nucleus in the trophoblast cell was indicative of inhibited endoreduplication [25]. Endoreduplication is required for rapid differentiation and intensive cell growth [26].

**Figure 1:**
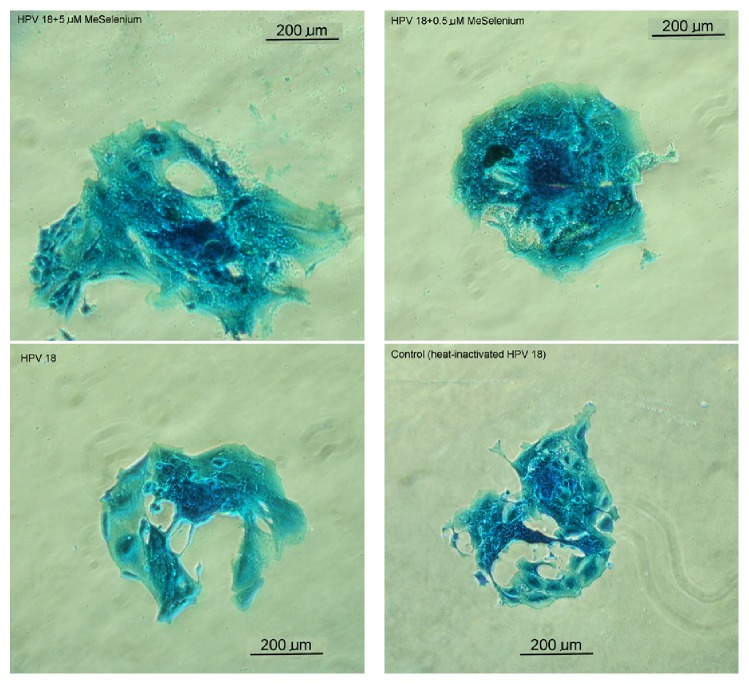
Implanted mouse trophoblasts cells after 48-hour exposure (at 37°C, 5% CO_2_ in air) to either (1) control medium with heat-inactivated HPV-18 HeLa lysate, (2) HPV-18 HeLa lysate, (3) HPV-18 HeLa lysate and 0.5 *μ*M selenomethionine (SeMet), or (4) HPV-18 HeLa lysate and 5.0 *μ*M SeMet. The stain used was Rose Bengal and Fast Green-based Spermac staining kit and images were taken using the Nikon Diaphot inverted microscope (400x magnification). The cell sizes for the images were as follows: (1) control, 515 *μ*, (2) HPV-18 HeLa lysate, 609 *μ*, (3) HPV-18 HeLa lysate with 0.5 *μ*M SeMet, 622 *μ*, and (4) HPV-18 HeLa lysate with 5.0 *μ*M SeMet, 915 *μ* at the greatest width.

In terms of the entire cell size, the HPV-18 exposed trophoblast cells demonstrated cell growth and hypertrophy (Figure 1) in the presence of both low (63651.1 ± 16106.9 sq·*μ*) and high (105038.0 ± 8802.0 sq·*μ*) SeMet concentrations when compared with the control (45620.5 ± 10440.3 sq.*μ*). Exposure to HPV-18 alone (54477.3 ± 10352 sq.*μ*) did not affect overall trophoblast cell dimension (*p* < 0.09). Trophoblast cell hypertrophy has been reported to be associated with activating F-actin cytoskeleton assembly correlated with migratory or invasive activity [27]. Although the nuclear size of HPV-18 exposed trophoblasts treated with the higher 5 *μ*M concentration SeMet remained unchanged, the mean cell dimension in this group was over 93% larger in size (Figure 1).

The presence of HPV-18 decreased (*p* < 0.005) trophoblast cell viability (13.3% live) when compared with the control (23.6%) after 48 hours of culture (Table 2). However, when SeMet was also present in the HPV-18 exposed groups, cell viability was sustained similar to the control group (*p* > 0.49). Furthermore, the percentages of apoptotic cells were lower when SeMet was present in the HPV-18 exposed groups. Although a small increase in necrosis was noted in the SeMet groups, the higher apoptosis in the HPV-18 only group yielded an overall higher cell viability in the groups with SeMet supplementation. There was no dose response observed based on the 2 concentrations of SeMet tested in this study.

Using the dual fluorescence stains procedure, the status of each compacted cell of the ICM layer was readily assessed as a colored point of light. The results (Table 3) showed that HPV-18 decreased ICM cell viability by over 60% when compared with the control (17.5 versus control 45.5% live). The addition of SeMet to the HPV-18 exposed ICM cells did not prevent cell death resulting from HPV-18. The percentages of apoptotic ICM cells were the highest in the HPV-18 with 5.0 *μ*M SeMet group indicating a slower demise of ICM cells in the higher concentration of SeMet group. In contrast to trophoblast cells, the addition of SeMet to the ICM cells did not have a positive response in terms of sustaining viability when challenged with HPV-18 exposure.

## 4. Discussion

HPV infection rates in spontaneous abortions of the first and second trimesters have been reported to be in the 50–70% range [2, 3]. The presence of HPV has been detected in the placentas derived from spontaneous abortions and preterm deliveries cases [2]. The origin of the HPV found in the placental cells was postulated to be circulating cell-free HPV DNA in the blood. Indeed, using the sensitive QIAamp circulating nucleic acid kit procedure and TaqMan technology, Mazurek and colleagues provided evidence of the circulating cell-free HPV DNA in blood plasma [28]. Recent evidence suggested that one of the etiologies of placental pathology was impaired trophoblast cell adhesion and trophoblast cellular necrosis in the HPV-infected placenta [5, 6, 17, 29]. Trophoblast cells are placental cells that participate in embryonic implantation and form critical cellular layers to facilitate fetal and maternal blood interactions in the pregnant uterus. Another group of cells from the ICM layer gives rise to the fetus. During placental inflammation, the HPV expressed oncogenes E5, E6, and E7 correlated with increased reactive oxygen species (ROS) and oxidative stress in the trophoblasts [2–5, 30–33].

The trace element selenium is a component of antioxidative selenoenzymes, Glutathione Peroxidase (GPx) and Thioredoxin Reductase (ThxRed), that decrease oxidative stress. Recent studies provided evidence that inorganic sodium selenite and organic selenomethionine (SeMet) protected cells from oxidative stress caused by peroxides and metabolic inhibitors such as antimycin and rotenone [12, 13]. SeMet is a selenium-containing amino acid that upregulates the 2 selenoenzymes GPx and ThxRed responsible for depleting ROS and reactive nitrogen species (RNS) thereby reducing oxidative stress [13, 14]. Other studies have shown the involvement of SeMet in altered cell signaling and inhibited gene expression of the proinflammatory cytokine IL-1*β* [34]. However, for in vitro studies of isolated cultured cells, the SeMet effects would most likely involve only oxidative reactions.

In the present study, the results showed that the addition of SeMet prevented nuclear shrinkage in the HPV-18 exposed trophoblasts. A shrunken nucleus in the trophoblast cell would be indicative of inhibited endoreduplication [24, 25]. The mechanism of action likely involved SeMet upregulating GPx and ThxRed to enzymatically catalyze ROS into inert molecules such as water possibly through transference of energy away from the reactive peroxides [35]. In this manner, SeMet protected the trophoblast nucleus from damage. Furthermore, SeMet had a hypertrophic effect on the trophoblast cells in terms of expanded cell size, even in the presence of HPV-18. Previous reports in specific cell types such as mammary epithelial cells showed that SeMet increased cell proliferation and cell viability [36]. This suggested that SeMet supplementation blocked HPV-18 mediated damaging effects on structural aspect of the placental cell. An obvious issue to address was whether or not SeMet would sustain cell viability in the presence of HPV-18.

The results showed that when SeMet was present in the HPV-18 exposed trophoblast cells, viability was sustained similar to the control cells. Furthermore, the percentages of apoptotic cells were lower when SeMet was present in the HPV-18 exposed groups. In the absence of SeMet supplementation, HPV-18 decreased trophoblast cell viability by 44%. This confirmed the protective role of SeMet in placental trophoblast cells. However, more studies are still needed to address another important group of cells associated with the trophoblast, namely, the ICM or embryoblast cells.

Dual fluorescence stain analysis showed that HPV-18 decreased ICM cell viability by over 60% when compared with the control. The addition of SeMet to the HPV-18 exposed ICM cells had no effect on blocking cell death. Moreover, apoptosis of ICM cells was the highest in the HPV-18 with 5.0 *μ*M SeMet group. This suggested that SeMet had a differential effect on cell viability that depended on the specific cell type. Previous studies have reported variable results of SeMet from cytotoxicity in lymphocytes and fibroblasts [37, 38] to protective effects in chondrocytes and BeWo trophoblasts [33, 39], hence reinforcing the need to study SeMet differential effects on placental cell types.

Limitations and precautions of the present study included the use of cultured cells which might generate a different response in the in vivo environment and the lack of pretreatment cell morphology assessment which would have required invasive cell fixing and staining procedures. Although studies showed SeMet upregulated selenoenzymes that reduced oxidative stress, a limitation here was that possible effects of SeMet affecting the stability of the HPV-18 gene fragments or cellular uptake were not evaluated as these effects were beyond the scope of the present study. It is recognized that the HPV oncogenes also affect other pathways involving p53 and Rb genes and these have been reviewed [40]. Nevertheless, the end result of HPV exposure was trophoblast cell death and SeMet could be a potential supplement for the prevention of HPV-related pregnancy losses. Further studies are needed to corroborate the present findings of SeMet treatment to abrogate HPV-related pathogenesis in the placenta.

## Figures and Tables

**Table 1:** Comparison of the area (mean ± S.E.M.) of each trophoblast nucleus or each trophoblast cell size after 24-hour exposure (at 37°C, 5% CO_2_ in air) to either (1) control medium with heat-inactivated HPV-18 HeLa lysate, (2) HPV-18 HeLa lysate, (3) HPV-18 HeLa lysate and 0.5 *µ*M selenomethionine (SeMet), or (4) HPV-18 HeLa lysate and 5.0 *µ*M SeMet. The ICM cells were not analyzed due to limitations in stained compacted cells.

Treatment groups	Number of cells (*n*)	Size of nucleus (*µ* ^2^)	Size of trophoblast (*µ* ^2^)
(1) Control	23	355.6 ± 35.9	45,620.5 ± 10,440.3
(2) HPV-18 HeLa lysate	28	253.8 ± 28.5^a^	54,477.3 ± 10,352.0
(3) HPV-18 HeLa lysate and 0.5 *µ*M SeMet	20	401.9 ± 47.8^b^	63,651.1 ± 16,106.9^a^
(4) HPV-18 HeLa lysate and 5.0 *µ*M SeMet	26	275.5 ± 23.1	105,038.0 ± 8,802.0^a,b^

^a^Different from the control (1) group (*p* < 0.05).

^b^Different from the HPV-18 HeLa lysate (2) group (*p* < 0.05).

**Table 2:** Day 6 in vitro implanted mouse embryos were exposed (at 37°C, 5% CO_2_ in air) for 48 hours to either (1) control medium with heat-inactivated HPV-18 HeLa lysate, (2) HPV-18 HeLa lysate, (3) HPV-18 HeLa lysate and 0.5 *µ*M selenomethionine (SeMet), or (4) HPV-18 HeLa lysate and 5.0 *µ*M SeMet. The percentages of live, apoptotic, and necrotic trophoblast cells were assessed using bisbenzimide and propidium iodide dual-stain epifluorescence analyses.

Trophoblast cells group	Total cells (*n*)	Number of viable cells (%)	Number of apoptotic cells (%)	Number of necrotic cells (%)	Number of total nonviable cells (%)
(1) Control group	64	17 (23.6)	47 (76.4)	0 (0)	47 (76.4)
(2) HPV-18 HeLa lysate	134	16 (13.3)^a^	109 (79.8)	9 (6.9)^a^	118 (86.7)^a^
(3) HPV-18 HeLa and 0.5 *µ*M SeMet	187	51 (24.2)^b^	111 (62.2)^b^	25 (13.6)^a,b^	136 (75.8)^b^
(4) HPV-18 HeLa lysate and 5.0 *µ*M SeMet	132	35 (24.0)^b^	74 (58.7)^b^	23 (17.4)^a,b^	97 (76.1)^a,b^

^a^Different from the control (1) group (*p* < 0.05).

^b^Different from the HPV-18 HeLa lysate (2) group (*p* < 0.05).

**Table 3:** Day 6 in vitro implanted mouse embryos were exposed (at 37°C, 5% CO_2_ in air) for 48 hours to either (1) control medium with heat-inactivated HPV-18 HeLa lysate, (2) HPV-18 HeLa lysate, (3) HPV-18 HeLa lysate and 0.5 *µ*M selenomethionine (SeMet), or (4) HPV-18 HeLa lysate and 5.0 *µ*M SeMet. The percentages of live, apoptotic, and necrotic inner cell mass (ICM) cells were assessed using bisbenzimide and propidium iodide dual-stain epifluorescence analyses.

Treated ICM cells groups	Total cells (*n*)	Number of viable cells (%)	Number of apoptotic cells (%)	Number of necrotic cells (%)	Number of total nonviable cells (%)
(1) Control group	71	32 (45.5)	38 (53.2)	1 (1.3)	39 (54.5)
(2) HPV-18 HeLa lysate	365	41 (17.5)^a^	236 (55.3)	88 (27.2)^a^	324 (82.5)^a^
(3) HPV-18 HeLa and 0.5 *µ*M SeMet	236	44 (19.2)^a^	123 (50.6)	69 (30.1)^a^	192 (80.8)^a^
(4) HPV-18 HeLa lysate and 5.0 *µ*M SeMet	210	36 (20.6)^a^	148 (68.0)	26 (11.4)^a,b^	174 (79.4)^a^

^a^Different from the control (1) group (*p* < 0.05).

^b^Different from the HPV-18 HeLa lysate (2) group (*p* < 0.05).

## References

[1] Castle P. E. (2009). The evolving definition of carcinogenic human papillomavirus. *Infectious Agents and Cancer*.

[2] Gomez L. M., Ma Y., Ho C., McGrath C. M., Nelson D. B., Parry S. (2008). Placental infection with human papillomavirus is associated with spontaneous preterm delivery. *Human Reproduction*.

[3] Sarkola M. E., Grénman S. E., Rintala M. A. M., Syrjänen K. J., Syrjänen S. M. (2008). Human papillomavirus in the placenta and umbilical cord blood. *Acta Obstetricia et Gynecologica Scandinavica*.

[4] You H., Liu Y., Agrawal N. (2008). Multiple human papillomavirus types replicate in 3A trophoblasts. *Placenta*.

[5] Manavi M., Czerwenka K. F., Schurz B., Knogler W., Kubista E., Reinold E. (1992). Latent cervical virus infection as a possible cause of early abortion. *Gynakologisch-Geburtshilfliche Rundschau*.

[6] Hermonat P. L., Han L., Wendel P. J. (1997). Human papillomavirus is more prevalent in first trimester spontaneously aborted products of conception compared to elective specimens. *Virus Genes*.

[7] Blencowe H., Cousens S., Chou D. (2013). Born too Soon: the global epidemiology of 15 million preterm births. *Reproductive Health*.

[8] Foppoli C., De Marco F., Cini C., Perluigi M. (2015). Redox control of viral carcinogenesis: the human papillomavirus paradigm. *Biochimica et Biophysica Acta*.

[9] Zuo Z., Goel S., Carter J. E. (2011). Association of cervical cytology and HPV DNA status during pregnancy with placental abnormalities and preterm birth. *American Journal of Clinical Pathology*.

[10] Cho G., Min K.-J., Hong H.-R. (2013). High-risk human papillomavirus infection is associated with premature rupture of membranes. *BMC Pregnancy and Childbirth*.

[11] McDonnold M., Dunn H., Hester A. (2014). High risk human papillomavirus at entry to prenatal care and risk of preeclampsia. *American Journal of Obstetrics and Gynecology*.

[12] Rahmanto A. S., Davies M. J. (2011). Catalytic activity of selenomethionine in removing amino acid, peptide, and protein hydroperoxides. *Free Radical Biology and Medicine*.

[13] Watson M., van Leer L., Vanderlelie J. J., Perkins A. V. (2012). Selenium supplementation protects trophoblast cells from oxidative stress. *Placenta*.

[14] Walter R., Roy J. (1971). Selenomethionine, a potential catalytic antioxidant in biological systems. *Journal of Organic Chemistry*.

[15] Novotny L., Rauko P., Kombian S. B., Edafiogho I. O. (2010). Selenium as a chemoprotective anti-cancer agent: reality or wishful thinking?. *Neoplasma*.

[16] Fernandes A. P., Gandin V. (2015). Selenium compounds as therapeutic agents in cancer. *Biochimica et Biophysica Acta—General Subjects*.

[17] Hong L. J., Oshiro B. T., Chan P. J. (2013). HPV-16 exposed mouse embryos: a potential model for pregnancy wastage. *Archives of Gynecology and Obstetrics*.

[18] Chen S. S., Block B. S., Chan P. J. (2015). Pentoxifylline attenuates HPV-16 associated necrosis in placental trophoblasts. *Archives of Gynecology and Obstetrics*.

[19] Mincheva A., Gissmann L., zur Hausen H. (1987). Chromosomal integration sites of human papillomavirus DNA in three cervical cancer cell lines mapped by in situ hybridization. *Medical Microbiology and Immunology*.

[20] Meissner J. D. (1999). Nucleotide sequences and further characterization of human papillomavirus DNA present in the CaSki, SiHa and HeLa cervical carcinoma cell lines. *Journal of General Virology*.

[21] Gooding L. R., Aquino L., Duerksen-Hughes P. J. (1991). The E1B 19,000-molecular-weight protein of group C adenoviruses prevents tumor necrosis factor cytolysis of human cells but not of mouse cells. *Journal of Virology*.

[22] Rowland S. C., Jacobson J. D., Patton W. C., King A., Chan P. J. (2003). Dual fluorescence analysis of DNA apoptosis in sperm. *American Journal of Obstetrics & Gynecology*.

[23] Chan P. J., Corselli J. U., Jacobson J. D., Patton W. C., King A. (1999). Spermac stain analysis of human sperm acrosomes. *Fertility and Sterility*.

[24] Tolivia J., Navarro A., del Valle E., Perez C., Ordoñez C., Martínez E. (2006). Application of photoshop and scion image analysis to quantification of signals in histochemistry, immunocytochemistry and hybridocytochemistry. *Analytical and Quantitative Cytology and Histology*.

[25] Hinck L., Thissen J. P., Pampfer S., De Hertogh R. (2003). Effect of high concentrations of glucose on differentiation of rat trophoblast cells in vitro. *Diabetologia*.

[26] Yang V. S., Carter S. A., Ng Y. (2012). Distinct activities of the anaphase-promoting complex/cyclosome (APC/C) in mouse embryonic cells. *Cell Cycle*.

[27] Han J., Li L., Hu J. (2010). Epidermal growth factor stimulates human trophoblast cell migration through Rho A and rho C activation. *Endocrinology*.

[28] Mazurek A. M., Fiszer-Kierzkowska A., Rutkowski T. (2013). Optimization of circulating cell-free DNA recovery for KRAS mutation and HPV detection in plasma. *Cancer Biomarkers*.

[29] Wang J., Mayernik L., Armant D. R. (2007). Trophoblast adhesion of the peri-implantation mouse blastocyst is regulated by integrin signaling that targets phospholipase C. *Developmental Biology*.

[30] Boulenouar S., Weyn C., van Noppen M. (2010). Effects of HPV-16 E5, E6 and E7 proteins on survival, adhesion, migration and invasion of trophoblastic cells. *Carcinogenesis*.

[31] You H., Liu Y., Carey M. J., Lowery C. L., Hermonat P. L. (2002). Defective 3A trophoblast-endometrial cell adhesion and altered 3A growth and survival by human papillomavirus type 16 oncogenes. *Molecular Cancer Research*.

[32] Lai D., Tan C. L., Gunaratne J. (2013). Localization of HPV-18 E2 at at mitochondrial membranes induces ROS release and modulates host cell metabolism. *PLoS ONE*.

[33] Whiteside M. A., Siegel E. M., Unger E. R. (2008). Human papillomavirus and molecular considerations for cancer risk. *Cancer*.

[34] Cheng A. W. M., Stabler T. V., Bolognesi M., Kraus V. B. (2011). Selenomethionine inhibits IL-1*β* inducible nitric oxide synthase (iNOS) and cyclooxygenase 2 (COX2) expression in primary human chondrocytes. *Osteoarthritis and Cartilage*.

[35] Flohé L. (1978). Glutathione peroxidase: fact and fiction. *Ciba Foundation Symposium*.

[36] Miranda S. G., Purdie N., Osborne V., Coomber B. L., Cant J. P. (2011). Selenomethionine increases proliferation and reduces apoptosis in bovine mammary epithelial cells under oxidative stress. *Journal of Dairy Science*.

[37] Wu J., Lyons G. H., Graham R. D., Fenech M. F. (2009). The effect of selenium, as selenomethionine, on genome stability and cytotoxicity in human lymphocytes measured using the cytokinesis-block micronucleus cytome assay. *Mutagenesis*.

[38] Hazane-Puch F., Champelovier P., Arnaud J. (2014). Six-day selenium supplementation led to either UVA-photoprotection or toxic effects in human fibroblasts depending on the chemical form and dose of Se. *Metallomics*.

[39] Khera A., Vanderlelie J. J., Perkins A. V. (2013). Selenium supplementation protects trophoblast cells from mitochondrial oxidative stress. *Placenta*.

[40] Mighty K. K., Laimins L. A. (2014). The role of human papillomaviruses in oncogenesis. *Recent Results in Cancer Research*.

